# Transforming Bariatric Surgery Outcomes: The Pivotal Role of Enhanced Recovery After Surgery (ERAS) Protocols in Patient-Centered Care

**DOI:** 10.7759/cureus.52648

**Published:** 2024-01-21

**Authors:** Nabila N Anika, Mathani Mohammed, Abdullah Shehryar, Abdur Rehman, Sergio Rodrigo Oliveira Souza Lima, Yusra H Hamid, Ciara S Mimms, Shenouda Abdallah, Yash Sailesh Kumar, Muhammad Ibrahim

**Affiliations:** 1 Medicine and Surgery, Holy Family Red Crescent Medical College and Hospital, Dhaka, BGD; 2 General Surgery, Sudan Medical Specialization, Khartoum, SDN; 3 Internal Medicine, Allama Iqbal Medical College, Lahore, PAK; 4 Surgery, Mayo Hospital, Lahore, PAK; 5 Plastic Surgery, Bahia Hospital, Salvador, BRA; 6 Community Medicine, University of Khartoum, Khartoum, SDN; 7 Medicine, St. George’s University, Great River, USA; 8 Surgery, Sheikh Jaber Al-Ahmad Al-Sabah Hospital, Kuwait, KWT; 9 Medicine, Tbilisi State Medical University, Tbilisi, GEO; 10 Medicine, Allama Iqbal Medical College, Lahore, PAK

**Keywords:** bariatric surgery, perioperative care, systematic review, patient-centered outcomes, enhanced recovery after surgery (eras)

## Abstract

Bariatric surgery is a critical strategy in managing morbid obesity. Enhanced recovery after surgery (ERAS) protocols have revolutionized perioperative care in this field. This systematic review aims to synthesize current evidence on the impact of ERAS protocols on patient-centered outcomes in bariatric surgery. A comprehensive search across multiple databases was conducted, adhering to the Preferred Reporting Items for Systematic Reviews and Meta-Analyses (PRISMA) guidelines. Studies involving adult patients undergoing bariatric surgery and focusing on the implementation and outcomes of ERAS protocols were included. Data extraction and analysis emphasized patient recovery, well-being, and satisfaction. Eleven studies met the inclusion criteria. The review revealed that ERAS protocols are associated with reduced postoperative recovery times, decreased hospital stays, and enhanced patient satisfaction. Notably, ERAS protocols effectively reduced complications and optimized resource utilization in bariatric surgery. Comparative insights from non-bariatric surgeries highlighted the versatility and adaptability of ERAS protocols across different surgical disciplines. ERAS protocols significantly improve patient-centered outcomes in bariatric surgery. Their adoption facilitates a patient-focused approach, accelerating recovery and enhancing overall patient well-being. The findings advocate for the broader implementation of ERAS protocols in surgical care, emphasizing the need for continuous refinement to meet evolving healthcare demands. This review supports the paradigm shift toward integrating ERAS protocols in bariatric surgery and potentially other surgical fields.

## Introduction and background

Bariatric surgery has emerged as a key strategy in managing morbid obesity, offering significant benefits in terms of weight reduction and mitigating obesity-related health issues [1]. A notable development in this field is the introduction of enhanced recovery after surgery (ERAS) protocols. ERAS represents a fundamental shift in the way patients are cared for before, during, and after surgery. These protocols focus on expediting patient recovery and minimizing complications, marking a significant advancement in the surgical management of obesity [2]. The increasing reliance on bariatric surgery highlights the need for standardized care protocols such as ERAS, which are designed to optimize patient outcomes.

ERAS protocols, developed by the ERAS Society, advocate a comprehensive, multimodal strategy that encompasses various aspects of patient care [2]. This includes measures such as preoperative counseling, nutritional support, reducing the duration of preoperative fasting, employing effective pain management techniques, and promoting early physical activity after surgery. The success of these protocols relies on a team-based approach involving surgeons, anesthesiologists, nurses, and nutritionists working together to achieve rapid postoperative recovery, reduce hospital stays, and improve overall patient outcomes, impacting both physical healing and psychological well-being.

Recent systematic reviews and meta-analyses have underlined the effectiveness of ERAS protocols in bariatric surgery. They demonstrate significant improvements in safety, efficiency, patient recovery, and satisfaction, thereby emphasizing the importance of a patient-centered approach in bariatric surgical care [3,4].

The primary goal of this systematic review is to analyze and synthesize the current evidence on the impact of ERAS protocols on patient-centered outcomes in bariatric surgery. By focusing on aspects such as patient recovery, well-being, and satisfaction, we aim to provide a clear understanding of how ERAS protocols enhance the quality of care and outcomes for patients undergoing bariatric surgery.

To broaden our understanding of ERAS protocols and their wider applicability, this review also includes select studies from non-bariatric surgical fields. This approach allows us to draw comparative insights and highlight the adaptability and versatility of ERAS protocols across various surgical disciplines. These insights can help refine ERAS strategies for bariatric surgery. By examining the implementation and outcomes of ERAS protocols in both bariatric and non-bariatric contexts, we offer a comprehensive perspective on their effectiveness. This inclusion enriches our primary focus on bariatric surgery and underscores the potential of ERAS protocols in enhancing patient care and outcomes across a broad spectrum of surgical disciplines.

## Review

Materials and methods

PRISMA Standards of Care

In accordance with the Preferred Reporting Items for Systematic Reviews and Meta-Analyses (PRISMA) standards, our review adhered to a structured approach for collecting, analyzing, and presenting data. PRISMA is a set of guidelines developed to ensure the clarity, transparency, and integrity of reporting in systematic reviews and meta-analyses. These guidelines encompass a comprehensive checklist and a flow diagram. Key elements include a clearly defined rationale and objectives, explicit eligibility criteria for studies, systematic search strategies across multiple databases, meticulous study selection and data extraction processes, assessment of the risk of bias in individual studies, and thoughtful synthesis and analysis of collected data. By following PRISMA guidelines, we aimed to enhance the reproducibility, reliability, and validity of our systematic review findings. This adherence ensures that our review meets high standards for methodological rigor and reporting clarity, thereby providing a reliable and valuable contribution to the understanding of ERAS protocols in bariatric surgery.

Search Strategy

Our search strategy was developed following the PRISMA guidelines to ensure a systematic and comprehensive approach to identifying studies related to ERAS protocols in bariatric surgery. We extensively searched multiple electronic databases, including PubMed, Medline, Embase, and the Cochrane Library. We did not restrict by language or publication year to ensure all relevant studies were included. Keywords and MeSH terms such as 'bariatric surgery,' 'ERAS,' 'enhanced recovery after surgery,' and 'patient outcomes' were utilized. For example, we used Boolean operators such as 'AND' and 'OR' to combine these terms effectively, as in 'bariatric surgery AND ERAS' or 'enhanced recovery after surgery OR patient outcomes.’ Reference lists of identified articles were scrutinized for additional relevant studies, and our search was further enriched by examining clinical trial registries and conference proceedings for unpublished or ongoing research. An expert in medical information retrieval reviewed the final search strategy, ensuring it aligned with PRISMA guidelines and was comprehensive.

Eligibility Criteria

We have established multifaceted eligibility criteria to ensure a meticulous and relevant approach in this systematic review focusing on ERAS protocols in bariatric surgery. The inclusion criteria are designed to encompass a variety of study types, including peer-reviewed research articles, cohort studies, clinical trials, and systematic reviews with meta-analysis, ensuring a foundation of high-quality, evidence-based research. While the primary focus is on studies involving adult patients undergoing bariatric surgery to broaden our understanding and draw comparative insights, we also include select high-quality studies applying ERAS protocols in other surgical contexts, such as thyroidectomy, parathyroidectomy, liver surgery, and gynecologic oncology. These studies must specifically investigate the implementation and outcomes of ERAS protocols, including comparative analyses between ERAS and conventional surgical pathways. A crucial aspect of our review is the emphasis on patient-centered outcomes, such as recovery time, postoperative complications, length of hospital stay, and patient satisfaction. Only English studies are included to maintain consistency in language and interpretation.

Conversely, our exclusion criteria aim to uphold the precision and coherence of the review. We exclude studies not directly related to the implementation of ERAS protocols in bariatric surgery or the selected non-bariatric surgeries, ensuring the review remains focused on its primary objectives. Non-peer-reviewed articles, conference abstracts, posters, and unpublished works are also excluded to ensure source reliability. Additionally, any study that needs comprehensive data on the specified patient-centered outcomes or fails to compare ERAS and non-ERAS protocols is excluded. This is essential for maintaining the quality and relevance of our review. The inclusion of selected non-bariatric studies is justified by the need to understand the impact of ERAS protocols in a broader surgical context, potentially offering valuable insights for refining ERAS protocols in bariatric surgery. These studies are carefully chosen based on their methodological rigor and relevance to themes of rapid recovery and enhanced patient outcomes.

Data Extraction

The data extraction process for our systematic review of ERAS protocols in bariatric surgery and selected non-bariatric surgeries for comparative insights was meticulously structured to ensure accuracy and validity. This process was divided into two key stages to manage the information gathered effectively.

In the first stage, titles and abstracts of articles identified through our comprehensive search strategy were screened for preliminary relevance. Two independent reviewers assessed these abstracts, categorizing each as 'relevant', 'not relevant', or 'probably relevant'. This categorization was based on the article's pertinence to ERAS protocols, focusing primarily on bariatric surgery and considering selected non-bariatric surgeries for a broader understanding of ERAS application. The inclusion of non-bariatric surgeries in our review aims to draw comparative insights and enrich our knowledge of ERAS protocols across different surgical disciplines.

A detailed review of the full-text articles was conducted during the second stage. This involved two independent reviewers extracting data using a standardized Microsoft Excel form tailored to capture a comprehensive range of essential information. This information included the study's authors, publication year, country of origin, participant demographics, study settings, and design. Particular attention was given to the specific ERAS protocols implemented, the outcome measures reported, and the key findings of each study, with a focus on patient-centered outcomes. The form also included sections to note the applicability of findings from non-bariatric surgeries to the bariatric context.

To ensure the integrity and consistency of our data extraction process, any discrepancies between reviewers were resolved by involving a third reviewer. This third reviewer facilitated discussions to reach a consensus, ensuring a rigorous and uniform assessment of the studies. This approach enabled us to conduct a comprehensive and accurate data analysis, maintaining the high methodological standards set for this systematic review. Incorporating non-bariatric surgeries while maintaining a primary focus on bariatric surgery is expected to provide a nuanced understanding of ERAS protocols' versatility and efficacy across different surgical contexts.

Data Analysis and Synthesis

The data analysis and synthesis for our systematic review of ERAS protocols in bariatric surgery, including select non-bariatric surgeries for broader comparative analysis, were conducted with a high degree of thoroughness and methodological rigor. Our approach primarily involved a qualitative synthesis, complemented by descriptive statistics where applicable, to ensure a comprehensive understanding of the data.

We qualitatively synthesized the gathered data, identifying common themes, patterns, and insights across the selected studies. This involved a detailed examination of the outcomes related to ERAS protocols, particularly emphasizing patient recovery times, complication rates, length of hospital stays, and patient satisfaction levels. Our qualitative synthesis approach was primarily chosen due to the heterogeneity in study designs, patient populations, and outcome measures, which made a quantitative meta-analysis not feasible. By employing thematic analysis, we were able to draw meaningful conclusions from the diverse body of literature included in our review.

The qualitative analysis was extended to include non-bariatric studies, allowing us to explore the adaptability and implementation of ERAS protocols across different surgical disciplines. This comparative examination aimed to highlight the similarities and differences in the efficacy of ERAS protocols, drawing insights into their optimization for bariatric surgery.

In addition to qualitative analysis, we utilized descriptive statistics to provide an overview of the data where it was consistent and comparable across studies. This included aggregating statistical measures such as means, standard deviations, and percentages. However, it is important to note that the variability in reporting standards and outcomes across the studies occasionally limited the scope of our quantitative analysis, impacting the consistency and depth of data synthesis.

By detailing these aspects of our methodology, we aim to offer a balanced and transparent view of our systematic review process. This approach ensures that readers are fully informed about the methods used and the rationale behind our analytical choices, thereby maintaining the integrity and validity of our review. The inclusion of non-bariatric surgeries in our analysis adds depth, offering a more holistic view of the potential and adaptability of ERAS protocols across different surgical specialties.

Results

Study Selection Process

Our comprehensive search across multiple databases culminated in the initial identification of a substantial number of articles. This rigorous search strategy yielded 123 articles potentially relevant to our systematic review of ERAS protocols in bariatric surgery, including select non-bariatric surgeries for comparative analysis. Upon the removal of duplicates, a total of 105 articles remained.

The titles and abstracts of these 105 articles were then meticulously screened for preliminary relevance to our research focus. This screening process led to the shortlisting of 47 papers, which appeared to be aligned with the objectives of our review and met the preliminary inclusion criteria.

After this initial screening, the full texts of these 47 articles were carefully examined to verify their eligibility based on our detailed inclusion and exclusion criteria. This thorough examination resulted in the selection of 11 articles that met all the requirements for inclusion in our systematic review. These articles included studies on ERAS protocols in bariatric surgery and critical non-bariatric studies that provided valuable comparative insights.

Additional studies that fulfilled our eligibility criteria were not found while reviewing the references of these selected articles. Therefore, the final number of articles included in our systematic review stood at 11.

**Figure 1:**
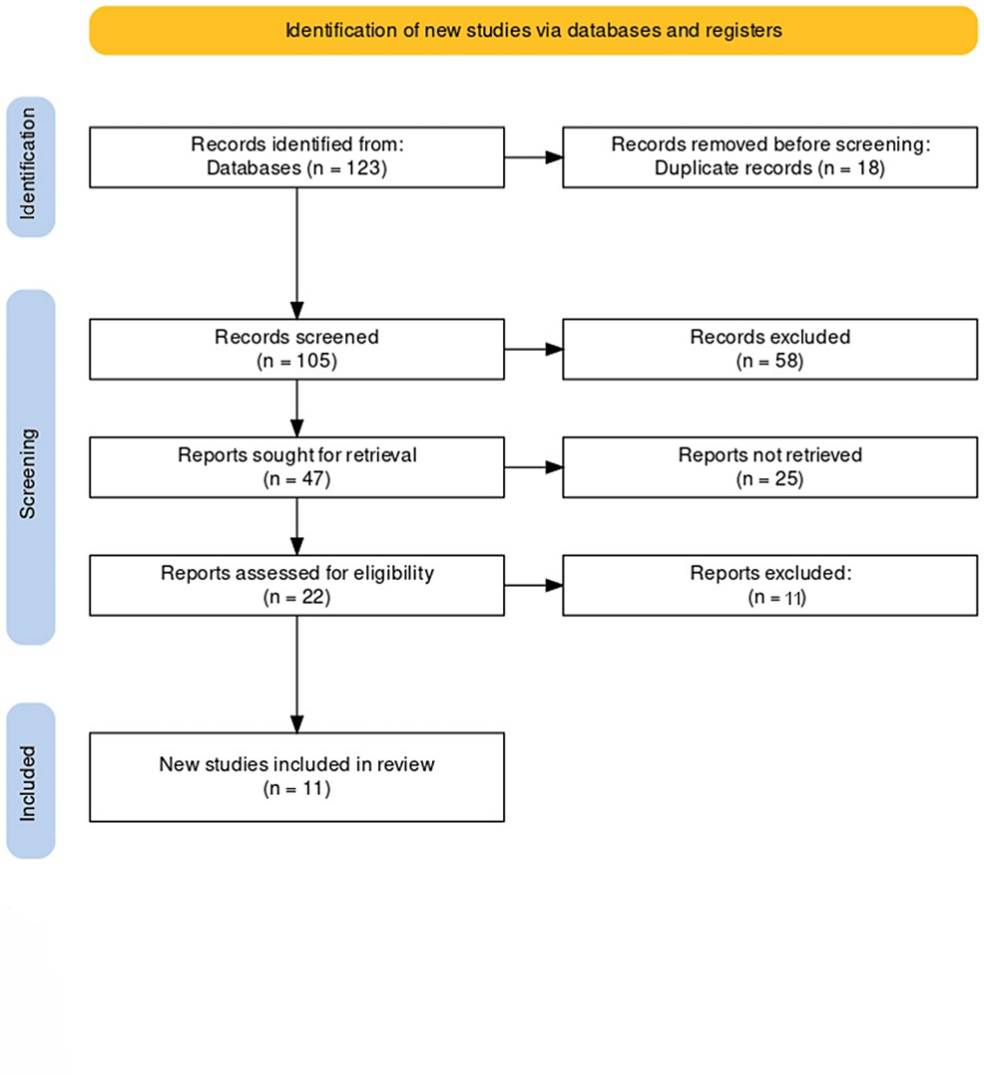
PRISMA flow diagram of the selection of studies for inclusion in the systematic review.

We have included a PRISMA flowchart to represent our study selection process clearly and visually (Figure 1). This flowchart illustrates the detailed steps taken from the initial identification of articles to the final selection of the studies included in our review, ensuring transparency and reproducibility of our research process.

Characteristics of the Selected Studies

The final selection for our systematic review consisted of 11 studies that rigorously met our inclusion criteria. These studies comprised diverse research designs, including retrospective reviews, systematic reviews with meta-analysis, observational cohort studies, and evidence-based reviews. Specifically, we included six studies focusing directly on bariatric surgery within the framework of ERAS protocols. Five studies provided comparative insights from other surgical disciplines, such as thyroidectomy, parathyroidectomy, liver surgery, and gynecologic oncology. This diversity in study design allowed for a comprehensive understanding of the implementation and impact of ERAS protocols.

Geographically, the studies were spread across various regions, reflecting a global perspective on the subject. Most of these studies were conducted in the United States and Europe, with significant contributions from the other areas. This wide geographical distribution enhances the generalizability of our findings.

The studies varied in sample size, study duration, and the specific aspects of ERAS protocols examined. Collectively, they covered a range of patient-centered outcomes, including recovery times, complication rates, lengths of hospital stays, and patient satisfaction levels. Each selected study's main findings and characteristics are meticulously detailed in the following table (Table 1). This table provides a concise overview of each study's key elements, facilitating a clear understanding of the collective body of evidence forming the basis of our systematic review.

**Table 1:** A summary of the studies included in this systematic review. ERAS: Enhanced Recovery After Surgery; BSTOP: Bariatric Surgery Targeted Opioid Prescribing Program; LOS: Length of Stay; AGB: Adjustable Gastric Banding; RYGB: Roux-en-Y Gastric Bypass; SG: Sleeve Gastrectomy; N/A: Not Available; RCTs: Randomized Controlled Trials; TAHs: Total Abdominal Hysterectomies

First Author	Year	Study Design	Study Technique	Study Duration	Sample Size	Conclusion
Tamara Díaz-Vico [5]	2022	Retrospective review	ERAS protocol vs. conventional pathway	Oct 1, 2016-Oct 31, 2018	ERAS: 173, Non-ERAS: 193	ERAS pathway for bariatric surgery was associated with less opioid usage, postoperative nausea, and vomiting, emergency department visits, and need for intravenous fluids without increasing length of stay, 90-day readmission, or rates of adverse effects.
Piotr Małczak [6]	2020	Analysis of the 15-element ERAS protocol	ERAS protocol adherence analysis	2009-2017	764 patients	Compliance with the ERAS protocol significantly affects morbidity and length of hospital stay after bariatric surgery. Further research is needed to identify the most impactful elements of the protocol.
Preet Mohinder Singh [3]	2017	Systematic review with meta-analysis	Meta-analysis of comparative trials between ERAS and conventional bariatric surgery	Published till June 2016	ERAS: 394, Conventional: 471	ERAS protocols for bariatric surgery allow a faster return to home but increase minor complication rates. High heterogeneity in current protocols calls for standardization. More randomized trials are needed for consolidation.
Jiajie Zhou [7]	2021	Systematic review and meta-analysis	Meta-analysis comparing ERAS protocols with standard care in bariatric surgery	Through May 2020	ERAS: 4964, SC: 3218	ERAS protocols in bariatric surgery effectively reduce hospital stays and the incidence of postoperative nausea and vomiting without increasing complications, readmissions, reoperations, or emergency room visits.
Jeffrey Silverstein [8]	2023	Retrospective review	Combined ERAS and BSTOP protocols in bariatric surgery	N/A	N/A	The combination of ERAS and BSTOP protocols in bariatric surgery significantly reduced the length of stay and opioid prescriptions at discharge without an increase in complication or readmission rates.
Kathleen E. Ackert [9]	2022	Retrospective review	ERAS protocol implementation for gynecologic oncology total abdominal hysterectomies (TAHs)	Implemented in 2018, duration not specified	Total: 183 (Non-ERAS: 59, ERAS: 124)	Implementing an ERAS protocol in gynecologic oncology for TAHs aimed to optimize patient outcomes, focusing on reducing opioid usage, length of stay, and cost. The challenges of implementing this large-scale protocol across a community network were also assessed.
Kevin Chorath [10]	2021	Systematic review and meta-analysis	Meta-analysis of studies on ERAS protocols for thyroidectomy and parathyroidectomy	N/A	3082 patients	ERAS protocols in thyroidectomy and parathyroidectomy surgeries significantly reduce hospital length of stay and costs without increasing complications or readmission rates.
Lyrics Noba [11]	2020	Systematic review and meta-analysis	Meta-analysis of RCTs and cohort studies comparing ERAS protocols with standard care in liver surgery	2008-2019	3739 patients (ERAS: 1777, Standard: 1962)	ERAS protocols in liver surgery are safe and feasible, reducing length of stay, complications, and hospital costs without increasing mortality and readmission rates. Further research is needed on overall compliance with ERAS protocols and its impact on outcomes.
Adetokunbo Obayemi Jr. [12]	2022	Evidence-based review	Review of ERAS protocol development and application in craniomaxillofacial surgery	N/A	N/A	ERAS protocols are effective in various surgical fields but require modification for craniomaxillofacial surgery. Their implementation can potentially reduce the length of stay, expedite recovery, reduce narcotic dependence, and decrease post-discharge complications.
Qinli Ma [13]	2021	Observational cohort study	Comparison of short- and long-term outcomes after three bariatric procedures (AGB, RYGB, SG) in a health plan network	Jan 1, 2006-Sep 30, 2015	95,251 adults (AGB: 34,240, RYGB: 36,206, SG: 24,805)	Interventions, operations, and hospitalizations were more frequent with AGB and RYGB than with SG. RYGB had the lowest risk for revision surgery, while SG and AGB had lower hospitalization and mortality risks than RYGB.
Jaime Dutton [14]	N/A	Systematic review	Review and meta-analysis of ERAS protocols in bariatric surgery	Since Jan 2015	N/A	ERAS protocols in bariatric surgery are associated with decreased length of stay (LOS) and increased early discharge rates. The review highlights the effectiveness of ERAS in streamlining care and improving recovery post-bariatric surgery.

Discussion

Impact of ERAS on Bariatric Surgery

Our systematic review rigorously evaluates the impact of ERAS protocols in the field of bariatric surgery, affirming their critical role in enhancing patient outcomes. The review uncovered a range of positive outcomes, notably reduced hospital stays and improved patient satisfaction. These findings align well with the existing literature, such as the work of Ljungqvist et al. [15], which documents similar benefits in various surgical settings. The consistency of our results with these established studies not only corroborates our findings but also reinforces the growing consensus regarding the efficacy of ERAS protocols.

ERAS: A Versatile and Universal Approach

The versatility of ERAS protocols, as highlighted in our review, resonates with the findings of Gustafsson et al. [16]. Their research into the application of these protocols across a spectrum of surgical procedures, not limited to bariatric surgery, underlines the adaptability and universal applicability of ERAS principles. This adaptability is crucial in the current healthcare environment, where surgical interventions are becoming increasingly complex, and patient-centricity is paramount.

Adopting ERAS protocols marks a significant shift toward more efficient, patient-focused care, a viewpoint echoed by Patel et al. [17]. Their advocacy for multimodal perioperative care strategies aligns closely with our findings, underscoring the potential of these protocols to enhance the overall surgical experience for patients. Furthermore, the economic aspect of ERAS protocols must be considered. The potential for cost savings, primarily driven by reduced postoperative complications and shorter hospital stays, as Varadhan et al. [18] have suggested, presents a compelling argument for their broader adoption.

Comparative Analysis with Non-bariatric Studies

While our systematic review primarily focuses on the impact of ERAS protocols in bariatric surgery, including non-bariatric studies, it is pivotal in enriching our understanding of these protocols in a broader surgical context. This comparative approach enables us to delineate the versatility and adaptability of ERAS protocols across various surgical disciplines, thereby providing a more comprehensive perspective on their effectiveness and application.

The non-bariatric studies included in our review encompass a range of surgical fields, such as thyroidectomy, parathyroidectomy, liver surgery, and gynecologic oncology. These studies contribute significantly to our analysis by highlighting the universal principles and diverse implementations of ERAS protocols. For instance, in thyroidectomy and parathyroidectomy surgeries, ERAS protocols have been shown to significantly reduce hospital length of stay and costs without increasing complications or readmission rates, similar to findings in bariatric surgery. Such parallels underline the efficacy of ERAS protocols in minimizing hospital stays and enhancing cost-effectiveness, irrespective of the surgical field.

Tailoring ERAS for Bariatric Surgery

Moreover, the implementation challenges and successes observed in non-bariatric surgeries offer valuable insights for refining ERAS protocols in bariatric surgery. For example, the study on gynecologic oncology reveals the complexities involved in applying large-scale ERAS protocols across different healthcare settings. Understanding these challenges can inform more effective and tailored application of ERAS protocols in bariatric surgery, ensuring better patient outcomes.

However, it is essential to note the distinct nature of bariatric surgery, which deals with a unique patient demographic and comorbidities. While the fundamental principles of ERAS protocols, such as early mobilization, pain management, and nutritional support, remain consistent, the specific strategies and interventions may require customization to address the unique needs of bariatric surgery patients.

Limitations and Future Directions

Despite the extensive scope of our review, it is essential to acknowledge its limitations. The variability in healthcare systems, as discussed by Lambert et al. [19], could impact the implementation and efficacy of ERAS protocols in different regional contexts. Additionally, our focus on English-language studies might have led to the exclusion of relevant research in other languages, as noted by other scholars [20], potentially limiting the comprehensiveness of our analysis.

Future research should aim to standardize ERAS protocols globally, as suggested by Maessen et al. [21]. This standardization would ensure uniform implementation of these protocols, enhancing their efficacy and applicability. Longitudinal studies, as proposed by other researchers [22], could shed light on the long-term impacts of ERAS protocols, providing a deeper understanding of their sustained benefits. Furthermore, integrating ERAS protocols with emerging surgical technologies, a frontier ripe for exploration [23], represents an exciting opportunity to revolutionize surgical care further.

Concluding Remarks

Including non-bariatric studies in our systematic review is not merely for comparative purposes but to foster a deeper understanding of the multifaceted nature of ERAS protocols. Collectively, these studies emphasize the adaptability of ERAS protocols across different surgical specialties while highlighting the need for specific adjustments in their application in bariatric surgery. This comprehensive analysis underscores the potential of ERAS protocols to revolutionize patient care in various surgical contexts, advocating for their broader adoption and continuous refinement.

This systematic review significantly contributes to the existing body of research on ERAS protocols, underscoring their vital role in enhancing patient outcomes in bariatric surgery. The findings advocate for the widespread adoption of these protocols, supporting a growing movement toward a standardized, patient-centered approach in perioperative care [24]. The implications of our study extend well beyond bariatric surgery, offering valuable insights and strategies applicable across a range of surgical disciplines. As the medical community continues to evolve, the insights gleaned from this review can serve as a benchmark for enhancing surgical care and patient outcomes in the years to come.

## Conclusions

Our systematic review robustly demonstrates the substantial impact of ERAS protocols on improving patient-centered outcomes in bariatric surgery. This underscores a critical paradigm shift toward more efficient, patient-focused surgical care, especially relevant in the context of the increasing prevalence of morbid obesity and the corresponding rise in bariatric surgeries. The implementation of ERAS protocols in bariatric surgery not only enhances recovery and patient satisfaction but also optimizes healthcare resources. Including non-bariatric studies in our review offers essential comparative insights, revealing the adaptability and broader applicability of ERAS protocols across various surgical disciplines. These insights from non-bariatric surgeries enrich our understanding, suggesting potential refinements and more general applications of ERAS protocols within bariatric surgery.

Our findings advocate for the widespread adoption of ERAS protocols, aligning with a global movement toward more holistic, patient-centered surgical care. This movement sets a new benchmark for excellence in the field, indicating the potential of ERAS protocols to revolutionize patient outcomes across different surgical disciplines. The comprehensive scope of our review, encompassing both bariatric and non-bariatric surgical applications of ERAS protocols, contributes to a richer and more nuanced understanding of their impact. It reinforces the potential for these protocols to enhance surgical care, advocating for their broader application and continuous refinement to meet the evolving demands of healthcare and patient needs.
